# Skin to skin interactions. Does the infant massage improve the couple functioning?

**DOI:** 10.3389/fpsyg.2015.01468

**Published:** 2015-09-25

**Authors:** Antonio Gnazzo, Viviana Guerriero, Simona Di Folco, Giulio C. Zavattini, Gaia de Campora

**Affiliations:** ^1^Department of Dynamic and Clinical Psychology, Sapienza University of RomeRome, Italy; ^2^Department of Pedagogy, Psychology, Philosophy, University of CagliariCagliari, Italy

**Keywords:** infant massage, couple adjustment, childbirth, parental stress, depressive symptoms

## Abstract

Transition to parenthood is a critical stage of life due to several changes the couple has to handle. A large body of studies described how transition to parenthood can be linked to the onset of depressive symptoms, as well as the perception of a low social support, and an increased stress, representing a risk for the early mother–baby relationship. Infant massage (IM) emerged as a helpful tool to improve maternal skills in interacting with the baby, and leading toward a decreasing of post-partum symptoms. However, a growing body of literature highlights that men also may experience post-partum diseases, representing an additional risk for the development of the baby. To date, no study observed the impact of the IM on both partners. The aim of the current qualitative research is to observe the impact of the IM on a single couple of parents at childbirth. Pre (Time 1) and post-intervention (Time 3) procedure has been established to observe the changes occurring over the time in the couple. In particular, each member of the couple filled out the EPDS, the BDI-II, the MSPSS, and the PSI-SF both at Time 1 and at Time 3. The treatment (Time 2) was represented by the IM training, and lasted 4 weeks. Findings revealed a decrease in depressive symptoms in both partners, as well as an improvement of their perception of stress related to parental role. No changes has been detected with respect to the perception of social support. The IM seems to be a helpful approach to prevent the establishment of pathological conditions in new parents. Although no direct measures on the child were used, the current qualitative data seem to suggest that the IM may represent a valuable tool to prevent the onset of early negative outcomes of the baby. Further investigations and empirical data are needed to improve the knowledge in this field.

## Introduction

Infant massage (IM) is a traditional care practice particularly widespread in Africa and South Asia (Field, 2000), and over the past decades also in Western countries (Underdown et al., 2013). IM can be defined as “a systematic touch by human hands, which stimulates the tactile sense of the infant” (Abdallah et al., 2013, p. 663). IM consists of traditional Swedish and Indian massage techniques, yoga and reflexology, and it can be applied by both mother and father on the child’s arms, legs, back, chest, belly, and face, using a vegetable odorless oil, and according to a standard sequence designed by McClure (2000). As mentioned by Underdown et al. (2013), IM may help to establish eye contact, as well as a sensitive tone of voice and touch which in turn may help both the development of baby’s ability in regulating emotions (Belsky, 2001; Fonagy et al., 2004) and the dyadic attachment relationship (Beebe and Lachmann, 2002; Slade, 2005; Tronick, 2007).

Several studies highlighted the benefits of IM performed by mothers or medical staff. Indeed, premature infants gained weight (Field et al., 2004, 2010; Diego et al., 2005; Ang et al., 2012) with an increase of 21–48% compared to control groups. Preterm babies – randomly assigned to a control or massage therapy group – also reached a significant increase in body temperature when receiving the massage (Diego et al., 2008). Other studies documented a lower pain responses following sensorial stimulation (Bellieni et al., 2002; Ludington-Hoe and Hosseini, 2005; Jain et al., 2006; Bellieni et al., 2007; Diego et al., 2009; Moyer-Mileur et al., 2012), weight gain (Ferber et al., 2002; Mainous, 2002; Liu, 2005; McGrath et al., 2007; Vaivre-Douret et al., 2009; Diego et al., 2014; Ahmed et al., 2015), less amount of energy expenditure compared to when they had not received the massage (Lahat et al., 2007), and shorter hospital stay (Vickers et al., 2004; Mendes and Procianoy, 2008; Vaivre-Douret et al., 2009; Field et al., 2010). Furthermore, from a neurological point of view, the effects of IM on the baby results positively influencing the Heart Rate Variability (Field et al., 2010; Smith et al., 2013), reducing the stress response, leading to a more rapid maturation of both visual function and brain electrical activity (Guzzetta et al., 2009, 2011) and to a better neurodevelopment outcome in psychomotor and mental development, compared to the long term effects on the babies of the control group (Procianoy et al., 2010).

Infant massage encourages early mother–child interactions through the promotion of maternal ability to understand the baby’s cues, leading toward a decrease in depressive symptoms (Onozawa et al., 2001; Glover et al., 2002; O’Higgins et al., 2008). Thus, the IM affects the mother, and in turn may support her relationship with the child, offering a unique opportunity to learn how to adjust each other’s emotional states and to improve the physical and emotional dyadic contact (Shai and Belsky, 2011).

## The Couple Functioning during the Transition to Parenthood

Transition to parenthood is an important and critical stage of life. Several changes occur with the need to find a new couple adjustment. Studies highlighted the presence of a strong decrease in couple satisfaction (Lawrence et al., 2008; Doss et al., 2009; Mitnick et al., 2009) and high level of parental stress (Meijer and van den Wittenboer, 2007; Lawrence et al., 2008; Bartolo et al., 2013). Other studies showed how becoming a parent may lead to a strong decrease in the level of partners’ adjustment concerning the relationship (e.g., Shapiro et al., 2000; Feeney et al., 2001), even among couples who show high level of adjustment in pre-pregnancy (Lawrence et al., 2008).

An increased body of literature stressed the importance of maternal post-partum mental health in affecting the quality of couple relationship. In particular, post-partum depression (PPD) – the higher maternal mental health risk at childbirth – it is a clinical condition affecting 10–15% of women, and is able to compromise both the dyadic and the family functioning. PPD is represented by the loss of interest in normal activities, by a greater tendency toward crying, feelings of guilt, anxiety, excessive sense of fatigue, lack of self-esteem, loss of concentration, excessive concern for the child or a lack of worry, panic attacks, suicidal impulses (Wisner et al., 2002).

Recent studies suggested that men also may experience PPD. A recent meta-analysis (Paulson and Bazemore, 2010) reported that PPD affects 23.8% of women and 10.4% of men. Although the clinical relevance of this phenomenon, just a few studies investigated the co-occurrence of depressive symptoms in the couple after the childbirth and throughout the first year of the baby’s life (Matthey et al., 2000; Dudley et al., 2001; Zelkowitz and Milet, 2001). Soliday et al. (1999) showed that the percentage of couples with at least one parent experienced PPD is between 32.6–47%. Zelkowitz and Milet (2001) highlighted that the 24% of couples with a woman suffering of PPD had also a man with PPD, as measured at 6 months of the baby’s life. Matthey et al. (2000) found the 53% of mothers who scored high on the Beck Depression Inventory (BDI; Beck et al., 1961) at 12 months of the baby had partners who also reached high scores. Soliday et al. (1999) reported that 19.6% of both parents reported the presence of depressive symptoms 1 month after the delivery, and about 4.7% of them also at 8 weeks after the delivery (Raskin et al., 1990). Escribà-Aguir et al. (2008), considering a sample of 687 women and 669 men, found a prevalence of depression in the 10.3% of women, and a 6.5% of prevalence in men, as assessed by the Spanish version of EPDS (Cox et al., 1987). The authors reported that among men whose partners were experiencing PPD, a prevalence of depressive symptoms occurred in 14.5% of them, while in women whose husband were experiencing PPD the prevalence was about 23.3%. In the study leaded by Bielawska-Batorowicz and Kossakowska-Petrycka (2006), and considering a sample of 80 men and their partners, with babies of 3 to 4 months old, the authors found that 27.5% of men received scores above the cut-off score of 13 in the EPDS, as well as for 31.2% of women. These results showed that depressive mood, after the birth of the child, is often experienced by men also, and sometimes by both partners. Similar results were reported in the study of Goodman (2008), where the EPDS was used along with other measures aimed to investigate parenting stress and mother/father–infant interactions. The study was conducted on 128 couples and their babies, and findings showed that 28% of mothers was depressed at 2 to 3 months post-partum, and about the 22% of their partners was experiencing the same condition. The authors found that the existence of maternal depression significantly increased the risk for fathers to experience a higher stress, depression, and a greater dysfunctional father–infant interaction. However, PPD can be recognized since the 4th month after birth. The first 3 months, even called *baby blues*, implicate several biological and hormonal changes which can be underlined to rapid mood variations. Thus, other factors should be considered before and after the baby blues. Research findings showed that the perception of a low social support during and after the baby blues phase, represent a substantial predicting factor for the emerging of PPD (Gao et al., 2009; Negron et al., 2013; Verreault et al., 2014).

Post-partum depression has been extensively investigated in women (Di Folco and Zavattini, 2015; Yim et al., 2015) and – as described above – little research on men highlighted the existence of this condition within the couple. Given this assumption, it could be argued that the presence of depression in both parents represent a higher risk for the developmental outcome of the baby. To date, just a few studies (Goudreau and Duhamel, 2003; Tandon et al., 2011; Petch et al., 2012) paid their attention to helpful interventions for both partners after childbirth. To the best of our knowledge, no study at all observed the impact of the IM taught to both partners. Given that, our clinical study aimed to observe whether the IM training could shape the adjustment of parents after childbirth. Our hypothesis is that the IM taught to both partners may reduce the depressive symptoms, the perceived parental stress, and increase the perceived social support.

## Clinical Case Description

A couple of parents with their first child, a baby boy of 5 months, were involved in our study. Barbara was 38 years old while Luca was 41 years old. Both parents belong to a high socioeconomic status and they are both graduates. The couple has been married for 5 years and after several attempts to have a baby, Barbara got pregnant during their fourth year of marriage. They first contacted us when their son Marco was 3 months old. The couple told us about their difficulties during the pregnancy, in particular they described their past failures in trying to have a baby and the fear they felt when they realized the risk of a premature birth during the 6th month of gestation. This event forced Barbara to stay in bed until the delivery.

The baby was born at term, through a vaginal birth and with the use of the epidural anesthesia. Marco weighed 2840 g at birth, he was 49 cm long and he did not show any physical problem. Although they described the delivery as “*good enough*”, they then reported a feeling of strong concern with respect to the care of their child. This emotional status probably represented the reason why they agreed to participate in a pre–post assessment on an IM class, indirectly expressing a request of help.

## Procedure and Instruments

Before the beginning of the study, each parents has been informed that the study was not reviewed by the Ethics Committee, and that the maximum risk involved in their participation was related to experience distress. They all signed a written informed consent form before the study begins, and they were informed about the chance to withdraw from the study in any moment. Parents signed a written informed consent form for their baby, and they were also informed that literature does not highlight any risk in participating in IM classes for the time being.

Once they agreed to participate, the couple was involved from the 4th month of the child’s life in four weekly meetings with a duration of 1 hour and half each, as required by the IM training. During these meetings, they were instructed by a certified massage instructor. Meetings were attended by both parents practicing on their child, following the massage sequences shown by the teacher on a doll and by using a odorless oil that facilitates the massage and allow the child to continue to feel the smell of the parent. In each meeting, it has been displayed a specific sequence for each part of the body that will be massaged (foots, legs, hands, arms, abdomen, chest, back, and face). Before the beginning, the instructor encourages parents to take time to relax, and invite parents to ask the child for permission to begin the massage, to interpret signals of availability/unavailability of their child. If the child’s behavior indicates an unavailability to start, the massage does not begin. During the practical part, parents are informed about some issues concerning the care of the child, such as recognizing different types of baby’s crying and different types of agitation of the newborn, in order to understanding the best time to begin the massage. During the massage the baby may cry and/or fidget. Thus, the parents are helped in exploring the meaning of these signals in order to feel more comfortable during the massage and to better help their baby.

Before and after the IM training, we administered a set of self-reports with the aim to assess changes occurring over the time. This assessment consisted of two phases. The first phase was at 4th months of the baby (T1; Pre-Test), while the second was at 5 months of the baby (T2; Post-test).

### Measures

The following measures were administered to both mother and father during the pre- (T1) and post-intervention (T2):

#### Edinburg Postnatal Depression Scale (EPDS; Cox et al., 1987)

The Edinburg Postnatal Depression Scale (EPDS) is a *self-report* questionnaire used to assess depression, and consisting of 10 items (on a 0–3 Likert scale), which investigate the presence and intensity of depressive symptoms, specifically: anhedonia, guilt, anxiety, fear or panic, sadness and crying, sense of failure, difficulty sleeping, thoughts getting hurt. The questionnaire does not investigate signs of depression such as tiredness and fatigue, as they are considered general effects of childbirth and the post-partum. The minimum and maximum score of the test were, respectively, 0 and 30. The authors suggested a cut-off of 12/13 for the detection of depressive symptoms in a clinical assessment, while a cut-off of 9/10 if the questionnaire is used for social surveys or for screening. The questionnaire was also validated in Italian (Benvenuti et al., 1999), using the cut-off of 8/9 and in current study we used this version. The instrument showed a good internal consistency tested using Chronbach’s alpha coefficient (0.79), and Guttman split-half coefficient (0.82) (Benvenuti et al., 1999).

#### Beck Depression Inventory-II (BDI-II; Beck et al., 1996)

The BDI-II is a self-report questionnaire for the assessment of symptoms and attitudes typical of depression. The questionnaire consists of 21 items (Likert scale 0–3). It provides a Total score and two dimensions of self-reported depression. The first dimension is the Somatic-Affective area, which concerns the manifestations of depression such as loss of interest, loss of energy, changes in sleep and in appetite, agitation and crying. The second one is the Cognitive area, which concerns the psychological symptoms of depression and the episodes of pessimism, guilt, self-criticism, and worthlessness. The scoring is done as following: 0–13 scores indicate no depressive content; scores between 14–19 a mild depression; scores between 27–29 a moderate degree of depression; and scores between 30–63 a severe depression. Adequate internal consistency (Cronbach’s alpha = 0.92) and evidence for convergent and discriminant validity were reported (Beck et al., 1996).

#### Parenting Stress Index-short Form (PSI-SF; Abidin, 1995)

The Parenting Stress Index-Short Form (PSI-SF) is a self-report used to assess the parenting distress related to parental role and it consists of 36 items about parents’ perceptions of the child’s behavior and attitudes about parenting. Responses are rated on a 5-point Likert scale, ranging from strongly agree to strongly disagree and they are clustered into three subscales [parental distress (PD), parent–child dysfunctional interaction (P-CDI), and difficult child (DC)] composed of 12 questions each, plus one scale to control the defensive answers. The subscale Defensive Responding (DR) permits to evaluate the parent’s attempt to answer, trying to minimize any problems, or stress related to the relationship with their child. The parents answer in a defensive manner to deny the fact that being a parent is difficult. On this scale scores of 10 or less could mean that the parent is trying to represent him/herself as very competent, or that the parent does not invest in the parental role. Specifically, the subscales of PD refers to distress related to parental role, such as impaired sense of parental competence, stress due to the restrictions on his/her life imposed by the parental role, marital conflicts, lack of social support, and depression symptoms. Regarding the subscale of P-CDI, this refers to parental perception of his child as a negative factor in his life, something that does not allow the onset of an appropriate parent–child relationship. Concerning the subscale of DC, this refers to some characteristics of the child’s behavior, related to the temperament that makes him as an easy or DC to manage. Finally, the Total score in PSI-SF concerns the stress experienced when the mother or the father are parenting. Construct validity was appropriate and reliability scores (Cronbach’s alpha) ranged from 0.88 to 0.93 on the fourth scales (Guarino et al., 2008).

#### Multidimensional Scale of Perceived Social Support (MSPSS; Zimet et al., 1988)

The Multidimensional Scale of Perceived Social Support (MSPSS) is a brief questionnaire that assess the perceived social support by 12 items on a 7-point Likert scale, rating the level of agreement/disagreement with respect to each statement. The items are designed to assess three dimensions of perceived social support and they refer to the figures of significant support. In particular, the three scales relate to family, friends, and to a significant other. The instrument showed good psychometric properties: the coefficient Cronbach’s alpha for the total scale is between 0.87 and 0.93, while for the subscales ranges between 0.81 and 0.98; with regard to the stability over the time, test–re-test correlation for the total scale is 0.85 and for the subscales ranges from 0.72 to 0.85 (Zimet et al., 1988).

## Results

### Edinburg Postnatal Depression Scale

Before the beginning of the training, Barbara told us her past fears related to the failed attempts to get pregnant and the difficulties experienced in carrying on the gestation. Then, Barbara filled out the EPDS and she reported a score of 10 during the pre-intervention phase (**Table 1**). The cut-off of 8/9 suggests the presence of depressive symptoms risk (Benvenuti et al., 1999). She reached high scores on those items related to anxiety, fear, panic, and worries for no good reason. In detail, she reported to feel “*so unhappy to cry a few times”.* A core feature of Barbara’s answers was represented by the high score to the item indicating “*thoughts about harming herself*”, which lead us to assume she was not just reporting baby blues symptoms or a low risk for PPD. In addition, her mood seemed to be closely related to the onset of a clinically relevant depressive syndrome. At the end of her participation at the IM classes, her EPDS’ score was 0. The post-intervention indicated the absence of depressive risk, accompanied by an absence of panic or anxiety without good reason, as well as an absence of unhappiness and thoughts of self-harming. From a clinical perspective, she seemed to be more responsive and involved with her child. She seemed warmer and more focused on child’s needs.

**Table 1:** Barbara’s self report scores in the pre- and post-intervention.

Measures	Pre-intervention	Post-intervention
	**Barbara**	
EPDS	10	0
BDI-II	5	2
PSI-SF Total Stress	49	36
PSI-SF Parental Distress (PD)	22	12
PSI-SF Parent–Child Dysfunctional Interaction (P–CDI)	13	12
PSI-SF Difficult Child (DC)	14	12
PSI-SF Defensive Responding (DR)	12	7
MSPSS Total	5.08	4.75
MSPSS Family	5.5	4.25
MSPSS Friends	3.75	4.25
MSPSS Significant Other	7	6

During our first meeting Luca appeared disinterested and “*a man of few words*”. However, and similarly to his wife, Luca reported a score of 10 at T1 (**Table 2**), indicating the presence of depressive symptoms. He reported feelings of anxiety and panic, difficulties to sleep because of his mood, and a tendency to self-blame when things went wrong. After the training, his score dropped to 2, indicating an improvement of his mood, with a substantial decrease on the items described above, except for his tendency to self-blame.

**Table 2:** Luca’s self report score in the pre- and post-intervention.

Measures	Pre-intervention	Post-intervention
	**Luca**	
EPDS	10	2
BDI-II	1	2
PSI-SF Total Stress	44	42
PSI-SF PD	20	18
PSI-SF P-CDI	12	12
PSI-SF DC	12	12
PSI-SF DR	10	12
MSPSS Total	6.17	6.17
MSPSS Family	7	7
MSPSS Friends	5.5	5.75
MSPSS Significant Other	6.5	6

#### Beck Depression Inventory-II

Barbara reached a score of 5 on the BDI at T1 (**Table 1**), while Luca reported a score of 1 (**Table 2**). Both scores fall in a range (0–13) indicating a lack of depressive contents which is contradictory compared to the EPDS findings. However, a few details should be taken into account with respect to their answers. Barbara answered 1 on question about “Pessimism”, indicating a low but still present negative feeling about her own future, and also a score of 1 on “Loss of Energy”. Same score on “Concentration difficulty”, and a score of 2 on “Fatigue”. Luca reported a score of 1 on “Changes in appetite”, indicating more attention on his physical state, and an increased appetite with respect to the past. At T2, Barbara’s overall score was 2, with her reporting less pessimism and tiredness, and more concentration; while Luca’s overall score went from 1 to 2, associated to a greater sense of fatigue.

#### Parenting Stress Index-short Form

Barbara reported a PSI Total score of 49 (10° percentile) at the pre-intervention stage (**Table 1**), while Luca reported a score of 44 (10° percentile; **Table 2**). These scores highlighted how both parents described themselves as featured by a low perception of stress compared to those given by first time parents and represented by scores between the 15 and 80° percentile (Guarino et al., 2008). In details, Barbara’s individual subscales showed a score of 22 on the PD, a score of 13 on the P-CDI, a score of 14 on the DC sub-scale, and a score of 12 on the DR sub-scale. These scores revealed how she seemed to be comfortable in her parental role, and able to handle parenting tasks. She seemed to experience a positive relationship with Marco, featured by the perception of an “easy baby”, and by her tendency to avoid defensive responses.

At T1, Luca reported a score of 20 on the PD, a score of 12 on the P-CDI sub-scale, a score of 12 on the DC sub-scale, and a score of 10 on the DR sub-scale. These scores represent a feeling of comfort expressed by Luca, showing a sense of security in responding to the parenting tasks. However, he showed a greater tendency to respond in a defensive way compared to Barbara.

After the IM training, the scores of both parents showed a lower level of stress related to the parenting role, with Barbara reporting a total score of 36 and Luca a total score of 42. Considering the sub-scales, Barbara reported lower scores on each dimension, highlighting a lower attitude toward a defensive style, a lower PD, and a greater perception of her interaction with Marco. Given the self-report features, this result may be associated to the improvement in their parental skills, which in turn affect their perceived stress. Luca, instead, reported scores indicating his sense of comfort in the parental role and within the relationship with their child. His answers showed the lack of a defensive style and of the need to show the relationship with his child as positive.

#### Multidimensional Scale of Perceived Social Support

Barbara reported a score of 5,5 in the MSPSS’s Family sub-scale, which refers to the perceived social support from family. She also reached a score of 3,75 on the Friends sub-scale, and a score of 7 on the Significant Other sub-scale. Her MSPSS’ overall score is 5,08, (**Table 1**). These scores represent the perception of a greater social support received from both the family and the significant other compared to friends. Surprisingly, after the IM training, her scores decreased in each scale except for the Friends subscale, highlighting an overall lower perception of the perceived social support, while an increase on the social support experienced from friends.

Luca reported a scores of 7 on the Family sub-scale, a score of 5,5 on the Friends sub-scale, a score of 6,5 on the Significant Other sub-scale, with a Total score of 6,17 (**Table 2**). Luca described the family as the higher source of social support compared to other sources, and he also showed a greater perception of support provided by the environment.

## Discussion

The current single case study aimed to explore the possible effects of the IM training on a couple at childbirth. The main purpose of the training is to improve parental skills in touching the baby, which in turn may help their overall interaction with the child. According to the existing literature (Guzzetta et al., 2009, 2011; Procianoy et al., 2010), the IM taught to the mother improves the baby’s development promoting long-term benefits. Beyond this aim, our main goal was using IM as a way to promote the adjustment of parents after childbirth. In particular, our specific aim was to observe wheatear the IM training could promote positive changes with respect to the presence of depressive symptoms, to the perception of parenting stress, and to the perceived social support in both parents.

Regarding our first aim both partners showed a risk of depressive symptoms in the pre-intervention phase, as seen through the EPDS’ scores. These scores should be explained according to different perspectives. They can be the result of the hormonal changes occurring in the post-partum period, also known as the “maternity blues phase”, and represented by rapid mood changes, and by the tendency toward depressive feelings. Barbara and Luca described their fear during pregnancy in losing the baby, and their concern for a preterm delivery, which probably affected their subsequent adjustment to the perinatal period. Their previous failed attempts of having a baby, and their difficulties in handling a healthy pregnancy should be indeed considered as risk factors for the post-partum phase (Blom et al., 2010). Interestingly, these difficulties have emerged in both partners, suggesting how events related to expecting a baby and the maternal state during pregnancy affect the emotional functioning of the father as well, beyond the hormonal changes of the woman. Several studies (Goodman, 2004, 2008; Paulson and Bazemore, 2010) corroborate the idea that the couple works as a unit, and this is especially true in this phase, during which they seem to share – among other aspects – depressive symptoms. Although these difficulties may represent an obstacle in seeking for help, they decided to begin the IM training. On one hand, this choice showed how the feelings they experienced were disturbing, but on the other hand it also showed their desire in taking care of the baby, which represents a protective factor during this life stage. Their reciprocal adjustment to the perinatal stage also showed some differences with respect to the depressive symptoms. Barbara’s depressive symptoms emerged as a difficulty in regulating her emotional states, expressed by the tendency toward anxiety and depression. However, this features were associated to the ability to openly express her mood. Similarly, Luca showed a presence of depressive symptoms but with a greater tendency toward a defensive style (Figueiredo et al., 2007; Beebe et al., 2008). In addition to these results, BDI’s scores partially confirmed the presence of a depressive risk. However, some considerations should be reported. BDI showed how this couple was experiencing an overall sense of loss of energy, especially for Barbara, while a lack of depressive symptoms was found for Luca, partially disconfirming the EPDS results. One hypothesis might be that the BDI is a strong and reliable measure to detect depression in adults, but not enough adequate to explore the emerging depressive symptoms during the post-partum period (Affonso et al., 2000; Eberhard-Gran et al., 2002; Teng et al., 2005; Yonkers et al., 2009). A sense of fatigue and loss of energies are common and *normal* aspects after childbirth, whereas they can be seen as risk factors in other life’s stages. The interpretation of the BDI scores as specifically correlated to the couple’s mental health can represent a potential bias for our conclusion, and they worth to be considered with caution. However, at the end of the training – and according to those studies reporting the outcomes of the IM classes on mothers (Onozawa et al., 2001; Glover et al., 2002; O’Higgins et al., 2008) – the couple EPDS’ scores highlighted a drastic decrease, indicating the absence of depressive symptoms. These findings highlight how the IM training had an impact on the couple adjustment. However, given the length of the intervention and the lack of long term follow-up, the total absence of depressive symptoms should be seen as an index of positive change and not as a stable result.

Our second aim was related to the IM’s impact on parental stress. PSI’s results at T1 showed an overall sense of comfort for Barbara in being parent, accompanied by the pleasure to accomplish the parental tasks and by a general positive attitude within the relationship with the baby. After the IM training, Barbara showed an improvement in all these areas, reaching exceptionally positive scores related to a deep competence in her parental role. However, it should also pointed out that her score on the DR subscale might indicate the tendency toward the desire to show a positive attitude, instead of giving a reliable answer, as suggested by several authors (Paulhus, 2002; van de Mortel, 2008). Vice-versa, Luca showed a decreasing in the overall perception of his stress, as shown by the Total score, and the same was true for the stress related to the parental role. Differently, those areas concerning the baby – such as the stress related to the parent–child interaction and the perception of the child as difficult – did not changed. These findings seem to highlight how the IM may shape the personal sense of efficacy, which in turn affects the perception of stress. From this perspective, IM emerges as a tool, which may help the father in exploring the right way to be close and in touch with the baby. Thus, it is reasonable to observe an improvement on the perception of parental role and to a feeling of being competent. On the other side, the perception of the child as difficult vs. easy, or the distress in interacting with the baby, seemed to be related to the baby’s temperament, and to the interactions with him, which is something more concrete and objective compared to the parental role (de Campora et al., 2014; Cavanna and de Campora, 2015).

Lastly, our third aim referred to the perceived social support and to its change due to the IM training. Findings related to the MSPSS scores showed a decrease in Barbara’s perception and no changes in Luca’s view, which is somewhat peculiar. Indeed, previous literature (Gao et al., 2009; Negron et al., 2013; Verreault et al., 2014) stressed how the low perception of social support is at the same time a risk factor for PPD by itself, and the strongest predictor for the risk of PPD. Given that, we expected to observe an improvement in both these variables, as a result of the IM training. Our data did not confirmed this hypothesis. However, it should be pointed out that MSPSS does not distinguish between emotional and instrumental support. This characteristic limits the assessment of the social support as related to the depressive symptoms, and it should taken into account for further studies.

## Conclusion

The present study confirmed previous findings suggesting that the IM can be considered as a helpful intervention to decrease the symptoms of maternal PPD and to promote maternal ability to understand the baby’s signals (Onozawa et al., 2001; Glover et al., 2002; O’Higgins et al., 2008). The interesting outcome is the possibility to observe this improvement not only in the mother, as expected, but also in the father. Both participants showed improvements, which can be due to a direct effect of the massage on each parent in decreasing symptoms of depression, but also to an indirect effect of the maternal state on the emotional state of the father. Indeed, the decreasing risk of depressive symptoms in the mother may in turn affect the psychological state of the father. From this point of view, the IM training seems to work as a protective factor for the couple adjustment after childbirth. The improvement in parents’ mental health positively affects their ability in reading and responding to the baby’s signals, which in turn promote the child’s ability to regulate his own internal states (Fonagy et al., 2004).

Lastly, we observed the impact of the IM on the stress related to the parental role and on the perceived social support, variables often associated with PPD (Horwitz et al., 2007; Gao et al., 2009; Manuel et al., 2012; Negron et al., 2013; Verreault et al., 2014). In this study, the parents seemed to benefits from the use of IM training in terms of a decrease of the stress related to the new parental role, such as the perception of their parental responsibility or the feeling of being parents in a less restrictive way. Otherwise, the perception of social support does not seem to take advantage from the IM training, showing the importance to further investigate this area through different kind of measures.

In the present study, there are several limitations. First, the use of a single case approach limits the generalizability of our results and conclusions; in addition, the exploratory nature of this study does not allow comparisons of our findings against previous data. Despite these limitations, the case study still occupies a central position within the psychology research field, as it appears well-suited for the observation of a phenomenon within its real-life context. Indeed, many researchers do believe that single case studies are appropriate for the exploratory stage of an investigation (Yin, 2009), and that in any case they can be confirmed or disconfirmed by following replication studies. Secondly, the procedure does not provide the opportunity to control wheatear the observed changes were due to the IM or to the process of the time. However, previous research (Field et al., 2004, 2010; O’Higgins et al., 2008) findings suggest the positive impact of the IM during the post-partum phase. Deepen empirical researches and control groups should be used for this purpose. Thirdly, the use of self-report is a limitation by itself. Indeed, the parental availability to participate in our study, and their desire to provide a positive image of themselves, also through the replies to the self-reports, may represent a significant *bias* to the reliability of these data, and it should be considered for further investigations on this topic. Lastly, we did not assess the impact of the IM on the baby’s development. Additional measures and outcomes related to the baby should be taken into account and further considered.

With these limitations in mind, an important strength point should be highlighted. To date, no research observed the impact of the IM on both partners or on father only. According to a recent literature, and to significant social changes occurred in the caregiver roles, the investigation of the father role emerges as a central issue for the child’s development (Cowan et al., 2009; Fletcher, 2009; Di Folco and Zavattini, 2014; Di Folco et al., 2015). Literature (Diego et al., 2008; Field et al., 2010; Ang et al., 2012; Moyer-Mileur et al., 2012; Ahmed et al., 2015) showed how the infant can take advantage from the massage provided by the mother, but more investigations are needed on the effects of the massage performed by the father, and to its impact on his relationship with the baby.

In sum, the results discussed in our study describe the importance to provide early interventions for those cases at risk to experience PPD. The IM training seems to be a valuable way to protect the couple by the establishment of post-partum difficulties, and to improve their parental skills. The IM training provides an opportunity for parents to communicate with their child not just verbally but also through the quality of touch, the movement, the rhythm, and the bodily sensations (Underdown et al., 2013).

## Conflict of Interest Statement

The authors declare that the research was conducted in the absence of any commercial or financial relationships that could be construed as a potential conflict of interest.
